# Supplementary material for: Reusable Fe3O4/SBA15 Nanocomposite as an Efficient Photo-Fenton Catalyst for the Removal of Sulfamethoxazole and Orange II
Source: Nanomaterials (Basel). 2021 Feb 19;11(2):533. doi: 10.3390/nano11020533 (PMC7922933; doi:10.3390/nano11020533)
Supplement: Supplementary file 1 [file nanomaterials-11-00533-s001.zip › Supplementary files/20210131_SuplementaryFiles.docx]

| **a)**  **Orange II**  OII [633-96-5]  C_16_H_11_N_2_O_4_SNa  Sigma Aldrich  St. Louis, MI, USA  Azo dye  λ_max_ (nm) = 473 | **** | **b)**  **Methyl green**  MG [7114-03-6]  C_27_H_35_BrClN_3_·ZnCl_2_  Sigma Aldrich  St. Louis, MI, USA  Triarylmethane dye  λ_max_ (nm) = 633 |  | |
| --- | --- | --- | --- | --- |
| **c)**  **Cooper (II) phtalocyanine**  Cu(II)Pc [147-14-8]  C_32_H_16_CuN_8_  Sigma Aldrich  St. Louis, MI, USA  Phthalocyanine dye  λ_max_ (nm) = 754 |  | **d)**  **Reactive blue 19**  RB19 [2580-78-1]  C_22_H_16_N_2_Na_2_O_11_S_3_  Sigma Aldrich  St. Louis, MI, USA  Anthroquinone dye  λ_max_ (nm) = 593 | |  |
| **e)**  **Eriochrome® black T**  EBT  [1787-61-7]  C_20_H_12_N_3_NaO_7_S  Panreac Química  Barcelona, CT, ES  Azo dye  λ_max_ (nm) = 524 |  | **f)**  **Sulfamethoxazole**  SMZ  [723-46-6]  C_10_H_11_N_3_O_3_S  Sigma Aldrich  St. Louis, MI, USA  Sulfonamide  λ_max_ (nm) = 257 | |  |

**Figure S1.** Target compounds used in degradation experiments.

**Table S1.** Chemical characterization of the wastewater (N = 7)

| Parameter | Average, μ | Std. deviation, σ |
| --- | --- | --- |
| pH | 6.236 | 0.217 |
| TOC (mg L^-1^) | 4.719 | 2.409 |
| **Anions** |  |  |
| Chloride (mg L^-1^) | 19.239 | 31.968 |
| Nitrite (mg L^-1^) | 0.114 ^1^ | 0.159 |
| Nitrate (mg L^-1^) | 9.530 | 6.019 |
| Bromide (mg L^-1^) | 0.037 ^1^ | 0.098 |
| Phosphate (mg L^-1^) | 2.475 | 0.654 |
| Sulfate (mg L^-1^) | 12.426 | 3.438 |
| **Cations** |  |  |
| Lithium (mg L^-1^) | <LOD | <LOD |
| Sodium (mg L^-1^) | 16.515 | 4.974 |
| Ammonium (mg L^-1^) | 1.516 ^1^ | 0.897 |
| Potassium (mg L^-1^) | 4.706 | 1.191 |
| Magnesium (mg L^-1^) | 1.871 | 0.167 |
| Calcium (mg L^-1^) | 6.781 | 0.579 |

^1^ In some measures was obtained a value of < LOD, considered zero to calculate the average.


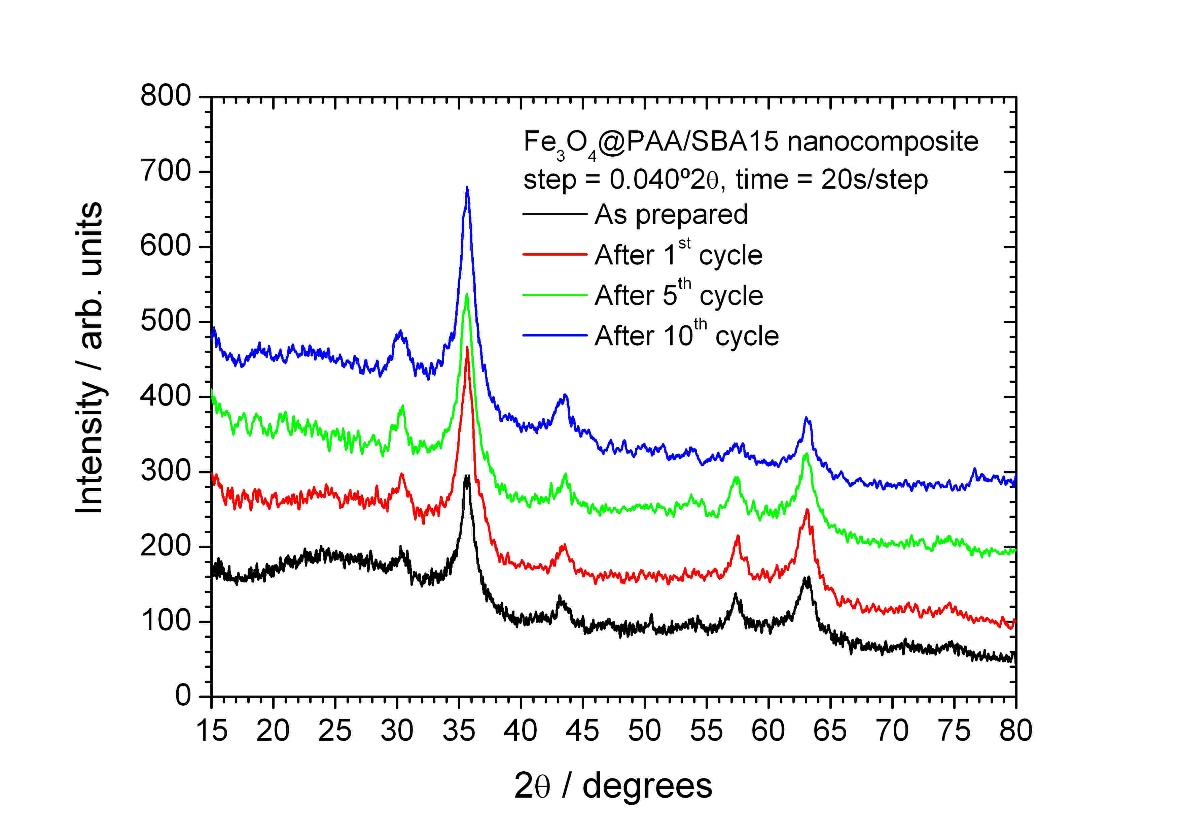


**Figure S2.** XRD characterization of fresh nanoparticles and after cycles 1, 5 and 10.

**Table S2.** Statistical parameters obtained from Box-Behnken optimization.

|  | Coefficient | σ² | F | p ^1^ |
| --- | --- | --- | --- | --- |
| Average | 0.116 | 0.119 |  |  |
| A: [Cat] | -0.161 | 0.222 | 0.53 | 0.488 |
| B: [H₂O₂] | 0.517 | 0.222 | 5.43 | 0.048 |
| C: pH | -2.806 | 0.222 | 160.03 | 0.000 |
| AC | 0.429 | 0.314 | 1.87 | 0.209 |
| BC | -1.245 | 0.314 | 15.75 | 0.004 |
| CC | 2.854 | 0.325 | 77.23 | 0.000 |

^1^ Considered significant when p < 0.05

**Table S3.** MicroTox® results of different treated effluents after OII removal at lab-scale experiments.

| Sample | EC_50,5_ | EC_50,15_ | EC_50,30_ |
| --- | --- | --- | --- |
| Raw water + OII | 9 | 5 | 4 |
| Effluent cycle 1 | 11 | 12 | 19 |
| Effluent cycle 5 | 14 | 17 | 18 |

**Table S4.** Optimization results for Fenton removal of OII using SBA15/Fe₃O₄@PAA

| [Cat] (mg Lˉ¹) | [H₂O₂] (mg Lˉ¹) | k (hˉ¹) | R² | %OII_rem_ |
| --- | --- | --- | --- | --- |
| 350 | 300 | 1.229 ± 0.062 | 0.98 | 97 |
| 500 | 200 | 1.026 ± 0.083 | 0.97 | 96 |
| 200 | 200 | 0.398 ± 0.023 | 0.98 | 72 |
| 350 | 100 | 0.121 ± 0.011 | 0.92 | 28 |

**Figure S3.** Kinetic constants (hˉ¹) [blue], TOC [green] and OII removal [orange] for lab-scale Fenton experiments.

**Figure S4.** Kinetic constants (hˉ¹) [blue], TOC [green] and SMX removal [orange] for lab-scale Fenton experiments.
