# Supplementary figures and images for: Reusable Fe3O4/SBA15 Nanocomposite as an Efficient Photo-Fenton Catalyst for the Removal of Sulfamethoxazole and Orange II
Source: Nanomaterials (Basel). 2021 Feb 19;11(2):533. doi: 10.3390/nano11020533 (PMC7922933; doi:10.3390/nano11020533)

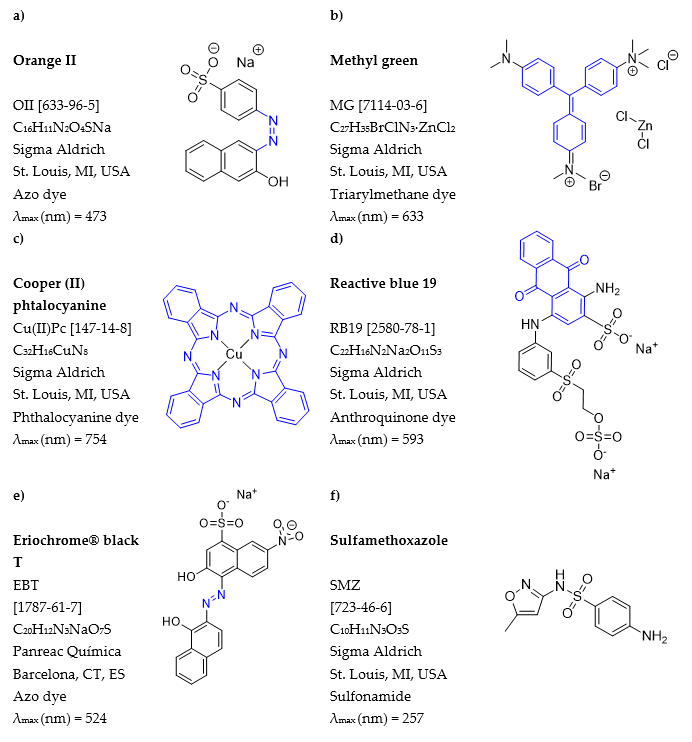

Supplement: Supplementary file 1 [file nanomaterials-11-00533-s001.zip › Supplementary files/Figure S1.PNG]

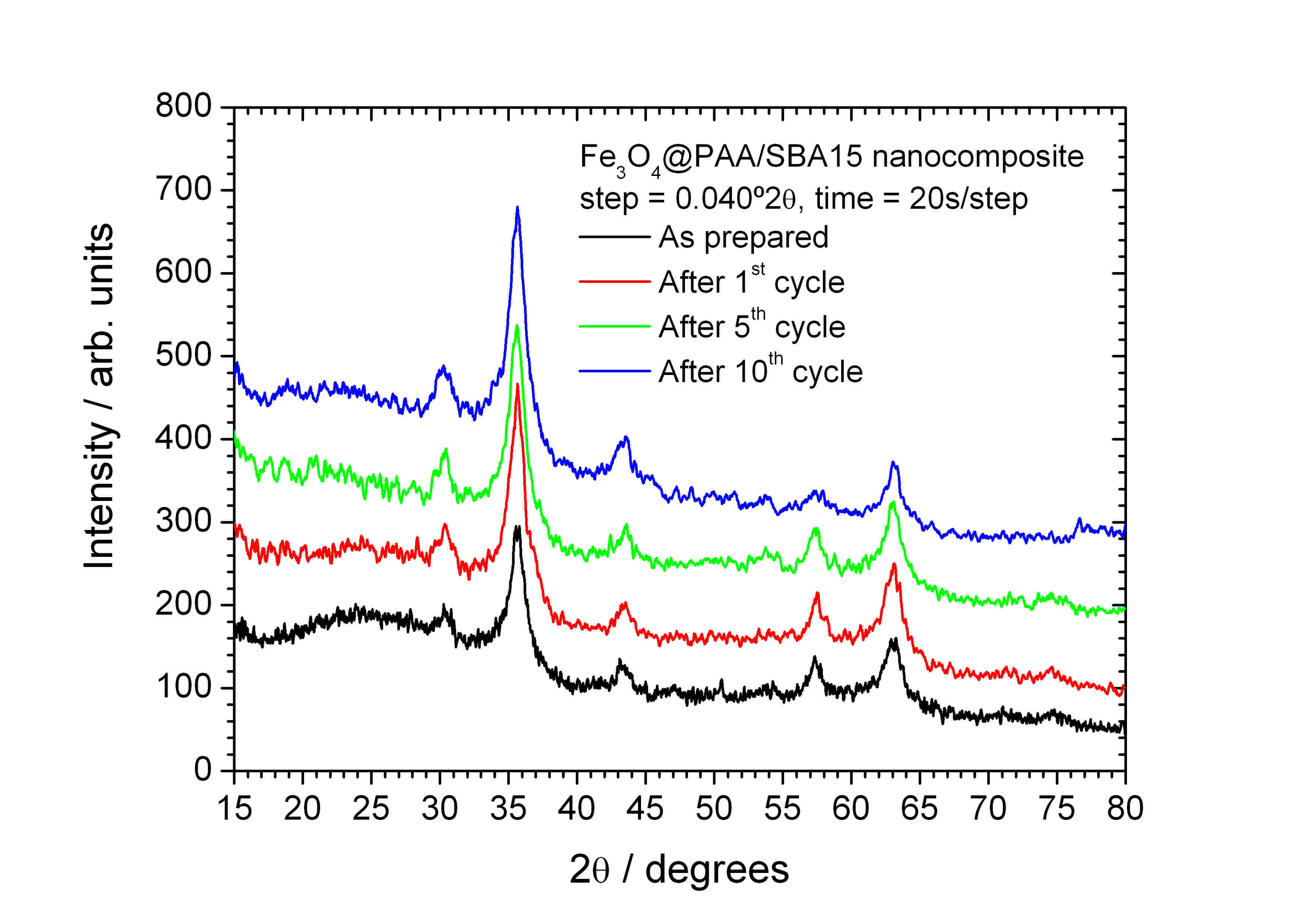

Supplement: Supplementary file 1 [file nanomaterials-11-00533-s001.zip › Supplementary files/Figure S2.JPG]
